# Interprovider variation of celiac disease testing in childhood chronic abdominal pain

**DOI:** 10.1186/1471-230X-13-150

**Published:** 2013-10-14

**Authors:** Bruno Pedro Chumpitazi, Krupa Mysore, Cynthia Man-Wai Tsai, Robert Jay Shulman

**Affiliations:** 1 Section of Gastroenterology, Hepatology, and Nutrition, Department of Pediatrics, Baylor College of Medicine, Houston, TX, USA; 2 Texas Children’s Hospital, 6701 Fannin Street, CCC 1010.03, Houston, TX, USA; 3 ARS/USDA Children’s Nutrition Research Center, Houston, TX, USA

**Keywords:** Celiac disease, Children, Abdominal pain, Serology, Variation

## Abstract

**Background:**

To determine within one tertiary care center: 1) the variation between providers in testing for celiac disease in children with chronic abdominal pain; 2) the characteristics of those children who were more likely to be tested, and 3) the prevalence of celiac disease in those evaluated.

**Methods:**

Retrospective review of children with a primary complaint of chronic abdominal pain referred to a tertiary care children’s hospital for pediatric gastroenterology evaluation over a 2-year period was conducted. Children with at least two visits and without an identified organic etiology for the pain were included.

**Results:**

160 children were evaluated by 16 pediatric gastroenterologists and one nurse practitioner. Celiac serologic testing was completed in 63 (39.4%) children. There was no significant variance in the frequency of celiac serologic testing between providers. Child age, gender, body mass index, and baseline gastrointestinal symptoms did not predict whether celiac serologic testing occurred, though Caucasians (P < 0.01) were more likely to be tested. Eighty-two (51.3%) children underwent either serologic testing and/or esophagogastroduodenoscopy. Four (4.9%, 95% CI: 1.6-11.3%) of the 82 tested were diagnosed with celiac disease.

**Conclusions:**

Though interprovider variation for celiac disease testing in children with chronic abdominal pain did not occur, a large number of these children were not evaluated for celiac disease. Children’s race/ethnicity but not their associated gastrointestinal symptoms predicted whether celiac testing was undertaken. In those tested, celiac disease was identified in a higher percentage than that expected in the general population.

## Background

Abdominal pain-related functional gastrointestinal disorders (AP-FGIDs) such as irritable bowel syndrome are prevalent, affecting up to 25% of school-aged children [1,2]. A child may meet criteria for an established AP-FGID in the absence of an inflammatory, metabolic, neoplastic or anatomic etiology, though identification of an organic process may depend on whether an evaluation is undertaken [2].

Celiac disease is an immunologically mediated response to gluten, resulting in an inflammatory enteropathy [3] and possibly causing gastrointestinal symptoms such as abdominal pain, constipation, or diarrhea [4]. These symptoms may overlap with those seen in children with AP-FGIDs, thereby challenging the clinician caring for these children to consider the diagnosis of occult celiac disease. A technical report on chronic abdominal pain in children suggests most children presenting to primary care do not require diagnostic testing, including celiac serologic testing [5]. However, pediatric gastroenterology practice guidelines for celiac disease in children suggest celiac disease testing in children with persistent gastrointestinal symptoms such as recurrent abdominal pain [6].

To our knowledge, the current practice with respect to celiac testing by pediatric gastroenterologists evaluating children with chronic abdominal pain is unknown, and significant variation in the frequency of testing may exist. Therefore, we sought to determine the frequency of celiac disease testing amongst providers at a tertiary care center for children who would otherwise be determined to have functional abdominal pain. We were further interested to determine the characteristics of those children who were more likely to be tested, and subsequently determine the prevalence of celiac disease in those evaluated.

## Methods

A retrospective chart review, of all children ages 4–18 years presenting to Texas Children’s Hospital for a new consultation for abdominal pain in the years 2006 and 2007, was completed. Those who experienced abdominal pain for a minimum of 3 months, returned for at least one follow-up visit after initial consultation, did not have celiac serologic testing performed by the referring primary care physician, and had abdominal pain entered into the electronic medical record as a primary diagnosis by the pediatric gastroenterologist were reviewed. Children who were subsequently diagnosed with an (potential) organic etiology (e.g. inflammatory bowel disease) as determined by the primary gastroenterologist, had any laboratory abnormalities suggesting an organic etiology, or who had resolution of their symptoms after starting on a proton-pump inhibitor were excluded. The remaining children essentially met criteria for an AP-FGID and were included. The Baylor College of Medicine Institutional Review Board approved this research study.

Demographic information, co-morbidities (Down syndrome, Turner’s syndrome, short stature, autoimmune hypothyroidism, Type I diabetes mellitus), and baseline gastrointestinal symptoms (vomiting, bloating, excessive flatus, belching, constipation, diarrhea, >10% weight loss) were captured from the medical record at the time of the initial consultation. Celiac serologic testing results (anti-tissue transglutaminase (TTG) IgA or anti-endomysial (EMA) IgA) and total serum IgA were obtained from the medical record. Serological testing was completed at various commercial laboratories as dictated by the children’s health insurance. Duodenal biopsy reports of children from the evaluated cohort undergoing esophagogastroduodenoscopy (EGD) were reviewed. Celiac disease was defined as having Marsh grade 1 or higher on duodenal biopsies and documented clinical improvement on a gluten-free diet.

Data were analyzed using SPSS version 19 (Somers, NY). Chi-square (or Fisher-exact when appropriate) testing was used to compare categorical data between groups. Fischer-exact testing was used to evaluate variance in testing frequency between providers. Student’s t-test was used to compare continuous parametric data between groups, while Mann–Whitney U was used to compare continuous non-parametric data between groups. Data are presented as mean ± standard error of the mean unless otherwise stated.

## Results

160 children were included and were evaluated by 16 pediatric gastroenterologists and one nurse practitioner (Table 1). One hundred fourteen (71.3%) were female, with mean age at the time of initial consultation being 10.5 ± 3.6 (range 4.1-17.8) years. Mean follow-up length was 8.0 ± 8.4 months. Self-identified race/ethnicities included: 66 (41.3%) Caucasian, 41 (25.6%) Hispanic, 10 (6.3%) African-American and 2 Asian (1.3%), with the remainder considered other or unclassified. Mean BMI% was 61.8 ± 30.4. Chronic abdominal pain was accompanied by constipation in 69 (43.1%), nausea in 39 (24.4%), vomiting in 38 (23.8%), diarrhea in 34 (21.3%), flatus in 19 (11.9%), bloating in 17 (10.6%), >10% weight loss in 16 (10%), and eructation in 6 (3.8%). One child had Down syndrome, and one child had Hashimoto’s thyroiditis with hypothyroidism.

**Table 1:** Evaluations and number of children with chronic abdominal pain undergoing celiac serologic testing by provider

**Provider**	**Children evaluated**	**Celiac serologic testing (%)**
Provider #1	32	12 (37.5%)
Provider #2	25	12 (48%)
Provider #3	25	12 (48%)
Provider #4	17	11 (64.7%)
Provider #5	15	7 (46.7%)
Provider #6	11	5 (45.4%)
Provider #7	8	4 (50%)
Provider #8	6	4 (66.7%)
Provider #9	6	4 (66.7%)
Provider #10	4	4 (100%)
Provider #11	2	0 (0%)
Provider #12	2	1 (50%)
Provider #13	2	1 (50%)
Provider #14	2	0 (0%)
Provider #15	1	1 (100%)
Provider #16	1	0 (0%)
Provider #17	1	1 (100%)

Celiac serologic testing was completed in 63 (39.4%). Providers did not have a statistically significant variance (P = 0.15) amongst themselves with respect to the frequency of celiac serologic testing (Table 1). This lack of statistically significant variance remained when comparing the frequency of serologic testing of the nine providers evaluating six or more of the included cohort. Age, gender, and baseline gastrointestinal symptoms did not predict whether serologic testing was completed (Table 2). 119 children had a self-identified race of Caucasian, African-American, Hispanic, or Asian. Of these children, Caucasian children were more likely than non-Caucasian children to undergo celiac serologic testing (35/66 vs. 12/53; P = 0.001). None of the children self-identified as African-American or Asian had celiac serologic testing completed.

**Table 2:** Demographic characteristics and gastrointestinal symptoms in children undergoing celiac serologic evaluation versus those who did not

	**Celiac serology evaluation (n = 63)**	**No celiac serology evaluation (n = 97)**	**P-value**
Age (years)	10.4 ± 3.9	10.6 ± 3.4	.74
BMI%	61.8 ± 28.3	64.2 ± 31.7	.64
Gender	Female: 40 (63.5%)	Female: 62 (63.9%)	.96
Vomiting	14 (22.2%)	24 (24.7%)	.71
Nausea	17 (27.0%)	22 (22.7%)	.54
Constipation	26 (41.3%)	43 (44.3%)	.70
Diarrhea	16 (25.4%)	18 (18.6%)	.07
Bloating	10 (15.9%)	7 (7.2%)	.08
Flatus	11 (17.5%)	8 (8.2%)	.08
Eructation	3 (4.8%)	3 (3.1%)	.59
Weight loss	7 (11.1%)	9 (9.3%)	.71

Either serologic testing or EGD was performed in 82 (51.3%) of the 160 children with both types of testing being completed in 20 (12.5%). Celiac serologic testing was abnormal in 3 children. Two subjects had elevated TTG IgA antibodies alone (EMA IgA was not tested), and one subject had both TTG and EMA antibody elevation. Two different subjects had low IgA levels (one clinically improved without further investigation whereas the other underwent an EGD that was negative for celiac disease). EGD with duodenal biopsies was completed in 39 (24.4%), including the three children with elevated TTG and/or EMA. Celiac disease ultimately was diagnosed in 4 (4.9%; 95% CI: 1.6-11.3%) of the 82 tested with all 4 having Marsh grade 3 histo-pathologic findings.

When comparing demographic information, BMI%, and GI symptoms of those undergoing celiac serologic testing and/or EGD between those found to have celiac disease vs. those who were not, 3 of 4 (75%) of those with celiac disease vs. 8 of 78 (10.2%) without celiac disease had flatus as GI symptom, P < .01. There were no significant differences seen in any of the other categories. None of the 4 children with celiac disease had weight loss.

## Discussion

To our knowledge, this is the first study to evaluate individual provider variation with respect to celiac testing in children with chronic abdominal pain. Though intra-center practice variation amongst providers did not occur, we found that less than half of these children underwent celiac serologic testing based upon the practice of seventeen pediatric gastroenterology providers at one tertiary care center. When combining both serologic testing and EGD, approximately half of the children were tested. This lack of uniform testing amongst the providers for the children evaluated suggests these providers are selective in their approach; therefore, recommendations regarding uniform celiac testing [6] in this population are not currently being followed.

Interestingly, we found that age, gender, body mass index, and gastrointestinal symptoms at the time of the initial consultation did not predict whether celiac serologic testing was completed. However, being Caucasian did increase the likelihood of serologic testing. We hypothesize that this may be due to the fact that providers recognize that certain high risk human leukocyte antigen alleles are found more commonly in the Caucasian population, with studies suggesting minority populations such as African Americans comprise only a small percentage of the celiac disease population in the United States [7,8]. Nevertheless, future investigation into whether race and/or ethnicity clearly impact the yield of celiac testing in children with chronic abdominal pain is needed to help guide providers caring for these patients.

There was a trend toward increased frequency of serologic testing (Table 2) in those children with diarrhea, bloating, and flatus. Interestingly, flatus was found to occur significantly more frequently in those with celiac disease. These symptoms are known to occur in celiac disease [9], and we note that given this trend toward testing in children with these symptoms there may be a higher frequency of celiac disease identified than that seen in a general population of children with functional abdominal pain. Further studies are needed to delineate associated symptoms which may increase the yield of celiac serologic testing in this population.

Our center’s findings with respect to the overall intracenter frequency of celiac testing in children with functional abdominal pain is supported by a previous study identifying that 57% of 122 children underwent celiac serologic testing when being evaluated at a tertiary care medical center for abdominal pain [10]. However, this study by Dhroove, et al. was limited in that children with chronic diarrhea, weight loss, and abdominal pain in particular locations were excluded. Though abdominal pain in certain locations and weight loss are considered “red flags” when evaluating a child with chronic abdominal pain [5], children and adults with these symptoms may still have a functional gastrointestinal disorder, particularly given a negative evaluation for an organic etiology [11-14]. As such, children with symptoms associated with celiac disease and functional gastrointestinal disorders were excluded in the Dhroove study. Potentially in concordance with these limitations, none of the studied children were subsequently found to have celiac disease, leading to an estimated prevalence of celiac disease in their population of chronic abdominal pain below that of the general population [7]. Nonetheless, the lack of universal celiac serologic testing supports our findings.

The prevalence of celiac disease in those tested in our study was higher than that found in a previous pediatric case–control study using celiac serology alone in the primary care setting [15]. The prevalence in this previous study in both those with chronic abdominal pain and controls was 1% (95% CI: 0-7%) similar to the estimated prevalence of celiac disease of 1% in the United States and European general population [3]. The difference in prevalence between our study and that of Fitzpatrick’s et al. may be due in part to the usage of EGD biopsy findings rather than serologic testing alone to make the diagnosis, and the exclusion of children with other organic contributors in our final study population (hence providing for a more selective group). This is supported by a recent systematic review and meta-analysis that found the pooled prevalence of celiac in adults with IBS in studies based upon biopsy-proved celiac disease to be 4.1% (95% CI:1.9-7.0) versus 1.63% (95% CI: 0.7-3.0) in those studies using serologic testing alone [16]. Moreover, our findings are further supported by a large epidemiologic screening study using primarily endomysial antibody testing with intermittent EGD confirmation which identified a 4.0% (95% CI: 2.99-5.20) prevalence of celiac disease in 1326 tested symptomatic (abdominal pain, constipation, diarrhea) children [4].

The primary limitation of this study is its retrospective nature. As such, initial medical evaluation, serologic testing laboratories, and endoscopies (e.g. number of duodenal biopsies taken) were not standardized. Another limitation is that children were not classified into a specific Rome III functional gastrointestinal disorder (e.g. irritable bowel syndrome vs. functional dyspepsia), though recent studies suggest this may be difficult to do even with prospective standardized criteria [17]. Finally, this is a single-center study; therefore, a future multicenter study is likely needed to confirm its results with respect to inter-provider variation.

One of the strengths of our study is its basis upon data generated in routine clinical practice at a tertiary medical center amongst numerous providers, which more likely reflects the actual experience in this setting and may help with generalizability of the findings. In evaluating the prevalence of celiac disease in those tested, another strength of this study is the use of endoscopic biopsies as part of the diagnostic criteria for celiac disease, as duodenal biopsy is the gold-standard for diagnosis [3]. All children with positive serologic testing underwent subsequent EGD; therefore, serologic false positives were not part of the prevalence determination. Serologic false negatives influencing the prevalence determination were decreased in our study as some of the children underwent EGD despite having negative celiac serology. In fact, one child in our cohort was identified with celiac disease despite having negative serologic testing.

## Conclusions

Though inter-provider variation for celiac disease testing in children with chronic abdominal pain did not occur, a large number of these children were not evaluated for celiac disease. We found that providers appear to be selective in their testing with children’s race/ethnicity, and trends for increased testing with those with diarrhea, bloating, and flatus. Nevertheless, given the higher prevalence of celiac disease found in children who would otherwise meet criteria for an AP-FGID in our study, we suggest consideration for celiac disease testing in this population at this time.

## Abbreviations

AP-FGID: Abdominal-pain related functional gastrointestinal disorder; EGD: Esophagogastro-duodenoscopy; EMA: Anti-endomysial antibody; TTG: anti-tissue transglutaminase antibody.

## Competing interests

The authors declared that they have no competing interests.

## Authors’ contributions

All authors read and approved the final manuscript. BPC participated in the design of the study, data collection, statistical analysis, and helped draft the manuscript. KM participated in the design of the study, data collection, and helped draft the manuscript. CMT participated in data collection and helped draft the manuscript. RJS participated in the design of the study and helped draft the manuscript.

## Authors’ information

BPC is an Assistant Professor of Pediatrics at Baylor College of Medicine. He is the Director of the Neurogastroenterology and Motility Program at Texas Children’s Hospital.

KM is currently a pediatric gastroenterology fellow at Baylor College of Medicine. She was a research assistant at Baylor College of Medicine during this project.

CMT is a research assistant at Baylor College of Medicine.

RJS is a Professor of Pediatrics at Baylor College of Medicine.

## Pre-publication history

The pre-publication history for this paper can be accessed here:

http://www.biomedcentral.com/1471-230X/13/150/prepub
